# Redo DAIR: The Game Is Seldom Worth the Candle

**DOI:** 10.3390/antibiotics12010018

**Published:** 2022-12-22

**Authors:** Ignacio Sancho, Iñaki Otermin-Maya, Jorge Gutiérrez-Dubois, Ignacio Aláez, Julián Librero, Maria Eugenia Portillo, Ángel Hidalgo-Ovejero

**Affiliations:** 1 Department of Orthopaedics and Trauma Surgery, Hospital Reina Sofía, 31500 Tudela, Spain; 2 Department of Orthopaedics and Trauma Surgery, Hospital Universitario de Navarra, 31008 Pamplona, Spain; 3 Campus de Arrosadía, Universidad Pública de Navarra (UPNA), 31008 Pamplona, Spain; 4 Department of Internal Medicine, Hospital Universitario de Navarra, 31008 Pamplona, Spain; 5 Instituto de Investigación Sanitaria de Navarra (IdiSNA), 31008 Pamplona, Spain; 6 Navarrabiomed, Complejo Hospitalario de Navarra—UPNA, 31008 Pamplona, Spain; 7 Department of Microbiology, Hospital Universitario de Navarra, 31008 Pamplona, Spain

**Keywords:** multiple debridements, prosthetic joint infection, second DAIR

## Abstract

Debridement, antibiotics and implant retention (DAIR) remains a commonly utilized technique in the treatment of acute prosthetic joint infections despite its inconsistent outcomes. The performance of a second DAIR after a failed first debridement is controversial as outcomes are uncertain and the final prognosis in the event of failure may be unfavorable. This study analyzes 84 cases of acute prosthetic (hip & knee) joint infection treated with DAIR between 2011 and 2020 at the same institution. In 12 failed cases, a second DAIR was performed, whose success rate was significantly lower than that of the first procedure (8% [95% CI, 0–38] vs. 57% [46–68]). Moreover, the ultimate outcome of the second failed DAIRs was unfavorable with eradication of the infection being achieved in none of the patients. Due to the high likelihood of failure and the potentially grim final prognosis following a second debridement, removal of the components should be considered.

## 1. Introduction

Debridement, antibiotics and implant retention (DAIR) is a viable treatment strategy for acute prosthetic joint infection (PJI) [1,2]. The technique is aimed at retaining a well-fixed implant and typically comprises an operative stage, where an attempt is made to decrease the intraarticular microbial burden through irrigation and debridement of the affected tissues, followed by a course of antibiotic treatment. Despite its highly variable success rates (33–88%) [3,4,5,6,7,8], the technique is associated with several advantages as compared with revision prosthetic surgery. These include lower morbidity rates, faster postoperative recovery and lower associated economic costs, among others.

Significant efforts have been made to improve the results of DAIR. Failure predictors related with the host, the Implantation/index surgery (IS) and the microorganisms involved have been established [1,2,5,9,10]. A series of tools have been developed in the last few years intended to help identify individuals where DAIR is more likely to fail [6,11,12], thus allowing a more efficient selection of candidates for the procedure. In cases associated with a high likelihood of failure, it would seem reasonable to opt for prosthetic revision surgery, which has been proven capable of healing the existing infection with higher success rates than DAIR [13,14,15]. 

There is also significant controversy as to what path to follow in the event of DAIR failure. In such a scenario, if the main goal is to eradicate an existing infection, it is possible to conduct a second DAIR procedure or proceed straight to a prosthetic revision surgery. Although some series of redo debridements have reported promising results [16], others show rather unfavorable success rates [7,17,18,19] (Table 1).

Additionally, according to some authors, patients where a DAIR procedure has failed could have a poorer final prognosis following a potential two-stage revision surgery [7,20,21,22]; the data was obtained mainly from knee replacement series. This finding has nevertheless been disproved by other TKA series [23,24,25,26]. At any rate, a poorer prognosis does seem highly likely in cases where not one, but two debridements have failed. Other negative consequences of successive failures include long periods of convalescence and their associated morbidity and mortality, and the medical costs inherent in multiple interventions. The factors outlined above suggest that the decision to perform a second debridement should be rigorously evaluated, carefully weighing all potential associated risks. 

Several authors have raised doubts about the advisability of performing repeat DAIR procedures [19,27]. In this regard, the latest International Consensus Meeting (ICM) on Orthopedic Infections, held in 2018, positioned itself in favor of prosthetic revision in the event of failed debridement [1]. The present study seeks to provide an answer to two important questions: 1. What is the success rate of second DAIR procedures? and 2. What is the final prognosis of patients where a second DAIR procedure has failed?

## 2. Materials and Methods

### 2.1. Study Design, Definitions & Criteria

A single-center retrospective cohort study was conducted at the Navarre University Hospital, a third-level hospital in the north of Spain, from January 2011 to December 2020. The implants analyzed were primary and revision total knee or hip arthroplasties.

Only debridements performed due to early acute PJIs (EAPJIs) and late acute PJIs (LAPJIs) were included. An EAPJI was defined as a PJI occurring within three months of prosthetic implantation whereas the LAPJI category included PJIs appearing beyond 90 days, with an abrupt onset of symptoms in patients with a hitherto normally functioning prosthesis [12]. All the DAIR procedures included were performed either within 90 days of the IS (in the case of EAPJIs), or within three weeks of the onset of symptoms (in the case of LAPJIs).

Exclusion criteria included patients where data were incomplete, those followed up for less than 12 months, and those subjected to arthroscopic debridements.

DAIR was considered to have failed when patients (1) had to undergo a subsequent procedure to control their infection, (2) required lifelong subsequent suppressive antibiotic therapy (SAT), or (3) subsequently died for reasons related to their PJI. 

Over the period under analysis, 84 acute PJIs were classified according to the European Bone and Joint Infection Society (EBJIS) definition [28] and were subjected to DAIR. The procedure was carried out within the timeframes mentioned above, depending on whether the infections were EAPJIs or LAPJIs. A redo DAIR procedure was attempted in 12 of the patients where the first DAIR failed to control the infection. This led to the classification of patients into a single-DAIR group (n = 72), made up of patients undergoing a single debridement, and a second DAIR group (n = 12), which comprised patients undergoing a repeat DAIR procedure. 

At the most recent follow-up, the patients’ status was defined according to the following categorization: (1) disease-free (successful treatment); (2) resection/fusion surgery; (3) SAT; and (4) persistent infection. The last three categories were considered to represent an unfavorable outcome. 

A database was created containing the clinical and demographic characteristics of the patients as well as a series of risk factors for the failure of DAIR. The analyzed variables included:

- Demographic & comorbidity risk factors: age, gender, American Society of anesthesiologists (ASA) classification, BMI (body mass index), Charlson’s comorbidity score, diabetes, immunodepression, renal or hepatic disease, anticoagulant or antiaggregant treatment and alcohol or tobacco abuse.
- Surgical predictors: indication for the IS (osteoarthritis [OA]/other), involved joint (hip/knee), type of prosthesis (primary/revision), and use of cement for implant fixation. In regard to the debridement, the time elapsed from the IS (EAPJI), from the onset of symptoms (LAPJI) and between the first and second DAIR procedure was measured. A record was also made of whether any mobile prosthetic components were revised.
- Clinical & laboratory findings: fever > 38°, drainage from the wound, culture-proven wound infection, presence of hematoma and/or fistulae. Laboratory parameters included C-reactive protein (CRP) levels, expressed in mg/dL, and white blood count (WBC) expressed in ×10^9^/L. The measurements analyzed were those taken closest in time to the DAIR procedure.
- Microbiology: tissue cultures, sonication and blood cultures. Microorganisms present were identified during the DAIR procedure, analyzing the number of positive cultures and any changes in the microbiological pattern between the first and second debridements. Cases where more than two different microorganisms were involved were classified as polymicrobial. *S. lugdunensis* was not considered part of the coagulase-negative staphylococcus (CoNS) group given its differential pathogenic behavior.

Institutional Review Board approval was obtained for using the patient’s data. Given the characteristics of the study, patient informed consent was not required. 

### 2.2. Diagnostic & Treatment

All IS’s and subsequent debridements were performed by the same surgical team. Team members remained largely unchanged throughout the period under analysis. 

Prior to the debridement surgery, all patients were subjected to blood tests. Joint aspiration and microbiological analyses were performed only in selected cases. Intraoperatively, at least five tissue samples (synovium, capsule and bone-implant interfaces) and one synovial fluid sample were routinely obtained for analysis. Extracted mobile components were in some cases sonicated. 

Using the pre-existing incision, a methodical and aggressive periprosthetic tissue debridement and a copious irrigation with at least 6L of saline were performed. In no instance were antibiotics delivered locally. Modular prosthetic components (femoral head, acetabular liner, and tibial insert) were not revised in all cases as such revisions were not common practice in our hospital during the first few years of the period analyzed. Aspirative drainage systems were used in all cases and removed at 48 h.

Immediately after surgery, patients received antibiotics targeted against fast growing planktonic bacteria. When a staphylococcal infection was present, such treatment included a combination of rifampicin with some other antibiotic (unless contraindicated), always in accordance with the recommendations of the internal medicine team and the results of the patients’ antibiogram. After the initial 1–2 weeks of intravenous treatment, patients were switched to oral antibiotic treatment, which lasted 6–12 weeks and included anti-biofilm agents. Both the surgical technique and the subsequent medical treatment were the same in the cases where the DAIR procedure was repeated. 

After a failed debridement, the decision whether to perform a redo DAIR or opt for a different strategy was always made by a multidisciplinary team on a case-by-case basis, evaluating whether the clinical and analytical signs of infection were still present and analyzing the patients’ characteristics and the microbiological findings. No specific decision-making algorithm was applied.

### 2.3. Statistical Analysis

A Clopper Pearson 95% confidence interval for binomial proportions was used to compare the success rates of all first DAIRs with those for all second DAIRs, as well as the ultimate outcomes of both procedures. 

A bivariate analysis was conducted to analyze the distribution of risk factors for DAIR failure. Categorical variables, expressed as absolute frequencies and percentages, were compared using the chi-squared test or Fisher’s Exact Test, as appropriate. Continuous variables were analyzed using Student’s t test or the Mann-Whitney U test, depending on whether their distribution was normal or not, respectively. Statistical significance was set at a *p* value < 0.05. Means and standard deviations were the parameters used to analyze normally distributed data, while medians and interquartile ranges were used for all other cases. The R v. 4.1.0 and R Core Team (2021) statistical software packages were used for the analysis [29].

## 3. Results

### 3.1. Patient Characteristics 

The characteristics of the patients subjected to DAIR (n = 84) in our hospital during the period under analysis who met the above-mentioned criteria can be seen in Table 2.

### 3.2. Success Rate of the Second DAIR Procedure

**Figure 1:**
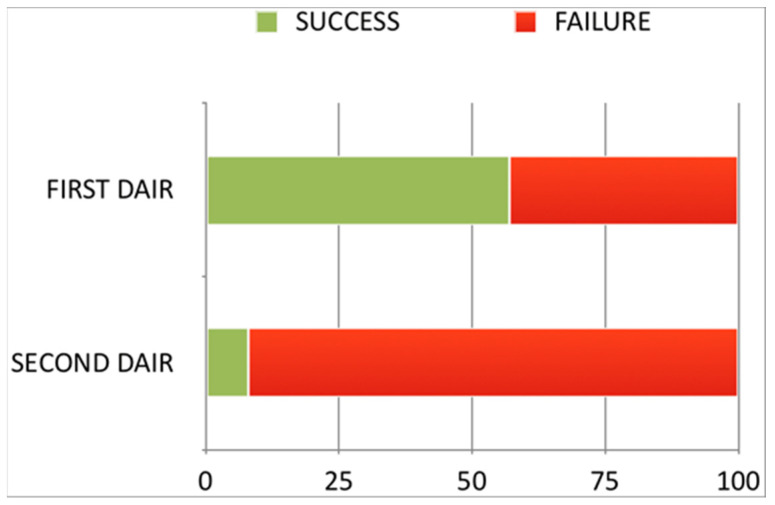
Success rates for the first and second DAIRs. DAIR: Debridement antibiotics and implant retention.

Overall, the effectiveness of DAIR in controlling infection was 57% (48/84) (95% CI: 46–68%) for initial debridements and 8% (1/12) for redo debridements (95% CI: 0–38%) (Figure 1).

### 3.3. Differences between the Single-DAIR and the Second-DAIR Groups

A comparison between the pre- and postoperative characteristics of patients in both groups—single and second DAIR—can be seen in Table 3. The second-DAIR group exhibited a higher frequency of IS’s performed for causes other than OA compared to the single-DAIR group (66.7% vs. 27.8%; *p* = 0.021). Differences were also observed regarding the types of implants debrided, with a lower proportion of total knee primaries in the second-DAIR group (8.3% vs. 47.2%, *p* = 0.027) but a higher proportion of both hip (33.3 vs. 15.3, *p* = 0.269) and knee (8.3% vs. 1.4%, *p* = 0.661) revision implants. Time to debridement in the group subjected to a redo DAIR was longer in the EAPJI group (44 vs. 29 days, *p* = 0.053).

In regard to comorbidities and symptoms, a higher incidence of liver failure (25% vs. 0%, *p* = 0.001) and of fever before the initial DAIR was observed in the second-DAIR group than in the single-DAIR group (66.7% vs. 27.8%, *p* = 0.021). 

As far as microbiological findings are concerned, no significant differences were found between the 11 failed redo DAIRs with regard to the species isolated during the first and the second debridement, or to those isolated at any further procedures those patients may have undergone. This means that there were no cases of late diagnosis or infections caused by newly introduced microorganisms.

### 3.4. Final Status Following Single vs. Repeat-DAIR

After failure of the second DAIR procedure, the disease could not be controlled in any of the patients, which means that 100% of patients experienced an unfavorable outcome (SAT/resection/fusion/persistent infection) (95% CI, 72–100%). This proportion was of 58% (95% CI, 37–78%) in the single-DAIR group. There were no amputations or PJI-related-deaths in either group. 

The final status of patients undergoing a failed single vs. second DAIR is shown in Table 4. Numbers and percentages of patients are shown for each ultimate outcome category (95% CI). 

## 4. Discussion

Several treatment alternatives exist for patients with PJI, including DAIR, one- stage or two-stage revision arthroplasty, SAT, fusion/resection surgery and amputation. DAIR has historically been the first-line treatment for PJI cases where the implant is stable, tissues are in good condition and the host is eligible for surgery. Indeed, the procedure allows preservation of the patient’s bone stock and is usually associated with lower morbidity and mortality rates, a lower economic cost and shorter recovery times. However, these theoretical advantages should be weighed against a greater difficulty to control infection and the procedure’s inconsistent results. 

When confronted with a DAIR procedure that fails despite an appropriate debridement, surgeons sometimes consider the possibility of performing a redo DAIR. However, if the rate of success of a single DAIR is moderate, the likelihood that the second debridement may succeed is usually rather low. The present series is no exception: all but one of the 12 repeat DAIRs carried out failed. This implies a success rate of 8% (95% CI, 0–38%), significantly lower than that reported for the single DAIR group (57% [95% CI, 46–68%]). This low success rate was also observed by other authors such as Lizaur et al. [18], who published a series of 24 redo DAIR procedures, all of which failed. These results would seem to support the recommendation by the 2018 Philadelphia consensus meeting that strong consideration should be given to removal of components in the failed single DAIR setting [1]. 

Wouthuyzen-Bakker et al. [16] however, reported very different results. In fact, in a study on 144 patients subjected to a second DAIR, these authors reported a success rate of 74.3%. Setting aside the sample size differences, the discordance between Wouthuyzen-Bakker et al. and the present study could be due to the fact that those authors only performed the redo DAIR procedure on patients with an “intact” soft tissue envelope, which could constitute a selection bias. The clinical and laboratory findings of our study (wound drainage, skin infection, hematoma, and discharging sinus tract) revealed that some of our patients presented with soft tissue problems, a common occurrence in this type of patient.

It could be hypothesized that patients undergoing a second DAIR procedure in our cohort were particularly complex in terms of their microbiological status, comorbidities, etc., and for that reason, the ultimate outcome of patients undergoing such procedures was more unfavorable that that of those undergoing a single DAIR. the large difference between the size of both cohorts (Single DAIR: n = 72 vs. Second DAIR: n = 12), a bivariate analysis was carried out of the incidence of risk factors for DAIR failure in each of them and no significant differences were found.

Although the second-DAIR group did exhibit a higher incidence of revision surgeries than the single-DAIR group—which might foreshadow a higher failure rate in that group—differences were not significant. It should also be noted that, although all three patients with liver failure were subjected to a second DAIR, neither the Charlson comorbidity score nor the ASA classification yielded markedly different values between the groups. Performance of a redo DAIR in many of our cases could have been prompted by the medical team’s belief that the procedure was preferable to a more aggressive prosthetic revision, which they believed should be reserved for more complex patients.

As already mentioned, not all patients where a debridement procedure has failed present with the appropriate soft tissue condition to undergo a second debridement and this could be a key factor in determining the procedure’s outcome. In our study, no significant soft tissue status differences were found between the groups, as evaluated based on the presence of hematomas, seromas, culture proven skin infection, or fistulas. Fever > 38° was found to be the only clinical variable with a significantly different distribution (66.7% vs. 27.8%, 95% CI, *p* = 0.021). In regard to time to the first debridement, it was only longer in the EAPJI group (44 vs. 29 days, *p* = 0.053). However, the difference was not significant. Nor was it for the mobile parts exchange (55.6% vs. 33.3%, 95% CI, *p* = 0.265)

As far as microbiological findings are concerned, although implant sonication was not routinely implemented in our department until the second half of the period under analysis, negative cultures amounted to 8.33% in both groups, a percentage unlikely to play an important role in the failure of the procedures. In the same vein, there were no discordances in the microbiological findings of the two groups or cases of antibiotic mismatch. One of the potential risks associated with a redo debridement is the intraoperative exposure to invading pathogens, i.e., the development of a second infection associated to the procedure. This circumstance did not arise in our series, and the microbiological profile of patients undergoing a redo DAIR remaining unchanged during follow-up.

We also considered the ultimate outcome in the failed second DAIR cohort. In these unfortunate cases of repeat failures, both the patients’ general health status as well as the specific status of their soft tissues could compromise a further revision surgery. There is also the risk of recalcitrant, and difficult-to-treat infections caused by multi-resistant pathogens, which may develop as a result of multiple surgeries and antibiotic treatments.

The unfavorable outcome criteria described above, including SAT and surgeries not involving joint reconstruction, must be taken into consideration when interpreting the results of the present study and comparing them with those of other series. Of the 24 failures in the single-DAIR group, 10 patients (41.2%) finally overcame their infection following revision surgery. Albeit low, the percentage was better than that for the second-DAIR group. Of the 11 failed second DAIRs, two patients underwent a failed revision surgery, five ended up on SAT and another were still bedeviled by a persistent infection at their last follow-up. The infection could not be eradicated in any of the 11 cases.

Finally, it is important to distinguish a second DAIR, which is performed following a failed first DAIR, from the so-called double DAIR, which is a two-stage DAIR procedure for acute PJI. Double DAIR, by definition, contemplates the performance of a second debridement as well as the local administration of high doses of antibiotics [30]. According to the available evidence, double DAIR appears to be a promising strategy, supported by disease control rates between 87 and 90% [31,32] and higher cost-effectiveness than one-stage DAIR. [33]

Despite its intrinsic limitations (retrospective design, small sample size, and no measurement of patient reported outcomes), we believe that the results of the present study, if taken in combination with those of similar studies, could be of assistance when making decisions regarding the treatment of patients with acute PJIs where a DAIR procedure has failed. Another strength of the study is that all the procedures were performed by the same medical-surgical team at the same hospital over a whole decade and that patients were subjected to long-term follow-up (minimum: 12 months, mean > 5 years (67.4 months).

Further studies on larger cohorts and containing an objective measurement of results and economic variables are required to provide more reliable data on the advisability of conducting redo DAIR procedures.

## 5. Conclusions

- Following an initial failed DAIR, a second DAIR has a low likelihood of success.
- After a second failed DAIR, the patients’ final prognosis is likely to be unfavorable.
- If an initial DAIR fails, it is advisable to switch to a prosthetic revision strategy.

## Figures and Tables

**Table 1:** Results of Second DAIRs in previous reported acute PJI cohorts.

Author	Data	Joint	N	Success (%)
Vilchez et al. [17]	2010	Hip & Knee	8	25
Lizaur et al. [18]	2015	Knee	24	0
Triantafyllopoulos et al. [19]	2015	Hip & Knee	15	53.3
Wouthuyzen-Bakker et al. [16]	2020	Hip & Knee	144	74.3

**Table 2:** Clinical characteristics of all patients who underwent DAIR (n = 84).

Demographics & Comorbidities		
Age	Mean (SD)	67.6 (11.8)
	min ≤ med ≤ max	16 ≤ 69 ≤ 85
	IQR(CV)	17 (0.2)
Gender	Male n (%)	56 (66.7%)
BMI (Kg/m^2^)	Mean (SD)	30.7 (5.8)
	min ≤ med ≤ max	19 ≤ 30 ≤ 51
	IQR (CV)	7 (0.2)
ASA score	I, n (%)	9 (10.7)
	II, n (%)	43 (51.2)
	III, n (%)	30 (35.7)
	IV, n (%)	2 (2.4)
Charlson score	0–3 n (%)	32 (38.1)
	4 or more, n (%)	52 (61.9)
**Surgical-related factors**		
Type of surgery n (%)	THA	32 (38.1)
	TKA	35 (41.7)
	RTHA	15 (17.9)
	RTKA	2 (2.4)
Cemented prosthesis	Yes n (%)	41 (48.8)
**PJI & DAIR variables**		
Type of PJI	EAPJI n (%)	72 (85.7)
	LAPJI n (%)	12 (14.3)
Mobile components exchange	Yes n (%)	44 (52.4)
Time from index surgery to DAIR (days) for EAPJI	Mean (SD)	37 (22)
	min ≤ med ≤ max	8 ≤ 29.5 ≤ 88
	IQR (CV)	25.8 (0.6)
Time from onset of symptoms to DAIR (days) for LAPJI	Mean (SD)	8.77 (5.9)
	min ≤ med ≤ max	2 ≤ 8 ≤ 20
	IQR (CV)	5.2 (0.7)
Time from first to second DAIR	Mean (SD)	31.8 (29.4)
	min ≤ med ≤ max	6 ≤ 19 ≤ 90
	IQR (CV)	31.8 (0.9)
Follow-up (months)	Mean (SD)	67.4 (33.6)
**Microbiology**		
Isolates n (%)	MSSA	24 (28.6)
	CoNS	23 (27.4)
	Polymicrobial	9 (10.7)
	Culture negative	7 (8.3)
	*S. dysgalactiae*	4 (4.8)
	*C. acnes*	3 (3.6)
	*S. lugdunensis*	3 (3.6)
	*Corynebacterium* spp.	2 (2.4)
	*P. aeruginosa*	2 (2.4)
	MRSA	2 (2.4)
	Others	5 (6)

ASA: American Society of Anesthesiologists; BMI: body mass index; CoNS: coagulase-negative staphylococcus; CV: coefficient of variation; DAIR: debridement antibiotics and implant retention; IQR: inter-quartile range; MRSA: methicillin-resistant staphylococcus aureus; MSSA: methicillin-sensitive staphylococcus aureus; OA: osteoarthritis; PJI: prosthetic joint infection; SD: standard deviation.

**Table 3:** Comparison between groups.

	Single DAIR, n = 72	Second DAIR, n = 12	*p* Value
Demographics & comorbidities			
Age (Median (IQR))	69 (60.75–77.00)	62 (57.50–72.00)	0.263 NonN
Gender = Male, n (%)	24 (33.3)	4 (33.3)	1.000
Charlson score; 4 or more, n (%)	44 (61.1)	8 (66.7)	0.963
ASA score (%)			0.313
I	7 (9.7)	2 (16.7)	
II	39 (54.2)	4 (33.3)	
III	25 (34.7)	5 (41.7)	
IV	1 (1.4)	1 (8.3)	
BMI (Kg/m^2^) (median (IQR))	31 (27.00–34.25)	28.50 (26.00–31.25)	0.161 NonN
Diabetes, n (%)	17 (23.6)	2 (16.7)	0.873
Inmunodepression, n (%)	6 (8.3)	0 (0.0)	0.665
Chronic renal failure, n (%)	7 (9.7)	2 (16.7)	0.829
Liver disease, n (%)	0 (0.0)	3 (25.0)	0.001
Antiplatelet drugs, n (%)	13 (18.1)	4 (33.3)	0.406
Anticoagulants drugs, n (%)	7 (9.7)	2 (16.7)	0.829
Smoking, n (%)	35 (48.6)	5 (41.7)	0.894
Alcohol, n (%)	15 (20.8)	2 (16.7)	1.000
Surgical related factors			
IS Indication other than OA, n (%)	20 (27.8)	8 (66.7)	0.021
Implant debrided, n (%)			0.042
THA	26 (36.1)	6 (50.0)	0.551
TKA	34 (47.2)	1 (8.3)	0.027
RTHA	11 (15.3)	4 (33.3)	0.269
RTKA	1 (1.4)	1 (8.3)	0.661
PJI & DAIR variables			
Cemented prosthesis, n (%)	48 (52.8)	3 (25.0)	0.141
Type of PJI: LAPJI, n (%)	9 (12.5)	3 (25.0)	0.484
Time from IS to 1st DAIR (days) (median (IQR)) for EAPJI	29 (21.50–42.50)	44 (34–72)	0.053 NonN
Time from clinical onset to 1st DAIR (days) (median (IQR)) for LAPJI	8 (4–8)	9 (8.50–14.50)	0.157 NonN
Mobile parts exchange, n (%)	40 (55.6)	4 (33.3)	0.265
Clinical & Laboratory findings			
Wound drainage, n (%)	39 (54.2)	9 (75.0)	0.301
Skin infection, n (%)	28 (38.9)	4 (33.3)	0.963
Hematoma, n (%)	24 (33.3)	5 (41.7)	0.815
Draining sinus tract	27(37.5)	5 (41.7)	1.000
Fever > 38°, n (%)	20 (27.8)	8 (66.7)	0.021
Serum CRP mg/dL (median (IQR))	6.75 (3–14.53)	13.50 (3.25–26.17)	0.424 NonN
WBC × 10^9^/L (median (IQR))	8.05 (6.57–10.53)	9.10 (5.50–13.22)	0.818 NonN
Microbiology			
Positive blood cultures n (%)	5 (6.9)	0 (0.0)	0.778
Isolated pathogens, n (%)			0.221
CoNS	22 (30.6)	1 (8.3)	
*Corynebacterium * spp.	2 (2.8)	0 (0.0)	
Culture negative	6 (8.3)	1 (8.3)	
*E. faecalis *	0 (0.0)	1 (8.3)	
*E. coli *	1 (1.4)	0 (0.0)	
*L. monocytogenes *	1 (1.4)	0 (0.0)	
*C. acnes *	3 (4.2)	0 (0.0)	
*P. aeruginosa *	2 (2.8)	0 (0.0)	
Polymicrobial	7 (9.7)	2 (16.7)	
*S. pneumoniae *	1 (1.4)	0 (0.0)	
*S. dysgalactiae *	3 (4.2)	1 (8.3)	
*S. lugdunensis *	2 (2.8)	1 (8.3)	
MRSA	2 (2.8)	0 (0.0)	
MSSA	20 (27.8)	4 (33.3)	
*S. marcesens *	0 (0.0)	1 (8.3)	
% of positive cultures (median (IQR))	60 (33–100)	100 (66.25–100)	0.129 NonN
Cultures positive 100%, n (%)	24 (33.3)	7 (58.3)	0.181

ASA: American Society of Anesthesiologists; BMI: body mass index; CoNS: coagulase-negative staphylococcus; CRP: c-reactive protein; CV: Cardiovascular; DAIR: debridement antibiotics and implant retention; IQR: interquartile range; IS: Index surgery; LAPJI: Late acute prosthetic joint infection; M: male; MRSA: methicillin-resistant staphylococcus aureus; MSSA: methicillin-sensitive staphylococcus aureus; NonN: Non normal; OA: osteoarthritis; PJI: prosthetic joint infection; RTHA: Revision total hip arthroplasty; RTKA: Revision total knee arthroplasty; SD: standard deviation; WBC: white blood cell.

**Table 4:** Final status in both groups following further treatment.

	Single DAIR Failures (n = 24)	Second DAIR Failures (n = 11)
Free n (%) (95%CI)	10 (41.2%) (22–63)	0 (0–28)
SAT n (%) (95%CI)	6 (25%) (10–47)	6 (55%) (23–83)
Resection or Fusion surgery n (%) (95%CI)	3 (12%) (3–32)	0 (0–28)
Recurrent infection n (%) (95%CI)	5 (21%) (7–42)	5 (45%) (17–77)

DAIR: Debridement antibiotics and implant retention; SAT: Suppressive antibiotic therapy.

## Data Availability

Datasets and R software package files are available from the corresponding author upon request.

## References

[1] Argenson J.N., Arndt M., Babis G., Battenberg A., Budhiparama N., Catani F., Chen F., de Beaubien B., Ebied A., Esposito S. (2018). Hip and Knee Section, Treatment, Debridement and Retention of Implant: Proceedings of International Consensus on Orthopedic Infections. J. Arthroplast..

[2] Osmon D.R., Berbari E.F., Berendt A.R., Lew D., Zimmerli W., Steckelberg J.M., Rao N., Hanssen A., Wilson W.R., Infectious Diseases Society of America (2013). Infectious Diseases Society of America. Diagnosis and Management of Prosthetic Joint Infection: Clinical Practice Guidelines by the Infectious Diseases Society of America. Clin. Infect. Dis..

[3] Cobo J., Miguel L.G.S., Euba G., Rodríguez D., García-Lechuz J., Riera M., Falgueras L., Palomino J., Benito N., del Toro M. (2011). Early prosthetic joint infection: Outcomes with debridement and implant retention followed by antibiotic therapy. Clin. Microbiol. Infect..

[4] Duque A.F., Post Z.D., Lutz R.W., Orozco F.R., Pulido S.H., Ong A.C. (2017). Is There Still a Role for Irrigation and Debridement With Liner Exchange in Acute Periprosthetic Total Knee Infection?. J. Arthroplast..

[5] Fehring T.K., Odum S.M., Berend K.R., Jiranek W.A., Parvizi J., Bozic K.J., Della Valle C.J., Gioe T.J. (2013). Failure of Irrigation and Débridement for Early Postoperative Periprosthetic Infection. Clin. Orthop. Relat. Res..

[6] Tornero E., Morata L., Martínez-Pastor J., Bori G., Climent C., García-Velez D., García-Ramiro S., Bosch J., Mensa J., Soriano A. (2015). KLIC-score for predicting early failure in prosthetic joint infections treated with debridement, implant retention and antibiotics. Clin. Microbiol. Infect..

[7] Urish K.L., Bullock A.G., Kreger A.M., Shah N.B., Jeong K., Rothenberger S.D., The Infected Implant Consortium (2018). A Multicenter Study of Irrigation and Debridement in Total Knee Arthroplasty Periprosthetic Joint Infection: Treatment Failure Is High. J. Arthroplast..

[8] Logoluso N., Drago L., Peccati A., Romanò D., Romanò C. (2014). Role for Irrigation and Debridement in Periprosthetic Infections. J. Knee Surg..

[9] Löwik C.A., Jutte P.C., Tornero E., Ploegmakers J.J., Knobben B.A., de Vries A.J., Zijlstra W.P., Dijkstra B., Soriano A., Wouthuyzen-Bakker M. (2018). Predicting Failure in Early Acute Prosthetic Joint Infection Treated With Debridement, Antibiotics, and Implant Retention: External Validation of the KLIC Score. J. Arthroplast..

[10] Löwik C.A.M., Parvizi J., Jutte P.C., Zijlstra W.P., Knobben B.A.S., Xu C., Goswami K., Belden K.A., Sousa R., Carvalho A. (2019). Debridement, Antibiotics, and Implant Retention Is a Viable Treatment Option for Early Periprosthetic Joint Infection Presenting More Than 4 Weeks After Index Arthroplasty. Clin. Infect. Dis..

[11] Wouthuyzen-Bakker M., Sebillotte M., Lomas J., Taylor A., Palomares E.B., Murillo O., Parvizi J., Shohat N., Reinoso J.C., Sánchez R.E. (2018). Clinical outcome and risk factors for failure in late acute prosthetic joint infections treated with debridement and implant retention. J. Infect..

[12] Shohat N., Goswami K., Tan T.L., Yayac M., Soriano A., Sousa R., Wouthuyzen-Bakker M., Parvizi J., On behalf of the ESCMID Study Group of Implant Associated Infections (ESGIAI) and the Northern Infection Network of Joint Arthroplasty (NINJA) (2020). 2020 Frank Stinchfield Award: Identifying who will fail following irrigation and debridement for prosthetic joint infection. Bone Jt. J..

[13] Durbhakula S.M., Czajka J., Fuchs M.D., Uhl R.L. (2004). Antibiotic-loaded articulating cement spacer in the 2-stage exchange of infected total knee arthroplasty. J. Arthroplast..

[14] Haleem A.A., Berry D.J., Hanssen A.D. (2004). The Chitranjan Ranawat Award: Mid-Term to Long-Term Followup of Two-stage Reimplantation for Infected Total Knee Arthroplasty. Clin. Orthop. Relat. Res..

[15] Sabry F.Y., Buller L., Ahmed S., Klika A.K., Barsoum W.K. (2014). Preoperative Prediction of Failure Following Two-Stage Revision for Knee Prosthetic Joint Infections. J. Arthroplast..

[16] Wouthuyzen-Bakker M., Löwik C.A., Ploegmakers J.J., Knobben B.A., Dijkstra B., de Vries A.J., Mithoe G., Kampinga G., Zijlstra W.P., Jutte P.C. (2020). A Second Surgical Debridement for Acute Periprosthetic Joint Infections Should Not Be Discarded. J. Arthroplast..

[17] Vilchez F., Martínez-Pastor J., García-Ramiro S., Bori G., Maculé F., Sierra J., Font L., Mensa J., Soriano A. (2011). Outcome and predictors of treatment failure in early post-surgical prosthetic joint infections due to Staphylococcus aureus treated with debridement. Clin. Microbiol. Infect..

[18] Lizaur-Utrilla A., Gonzalez-Parreño S., Gil-Guillen V., Lopez-Prats F. (2015). Debridement with prosthesis retention and antibiotherapy vs. two-stage revision for periprosthetic knee infection within 3 months after arthroplasty: A case–control study. Clin. Microbiol. Infect..

[19] Triantafyllopoulos G., Poultsides L.A., Zhang W., Sculco P.K., Ma Y., Sculco T.P. (2015). Multiple Irrigation and Debridements for Periprosthetic Joint Infections: Facing a Necessity or Just Prolonging the Inevitable?. J. Arthroplast..

[20] Sherrell C.J., Fehring T.K., Odum S., Hansen E., Zmistowski B., Dennos A., Kalore N. (2011). The Chitranjan Ranawat Award: Fate of Two-stage Reimplantation After Failed Irrigation and Débridement for Periprosthetic Knee Infection. Clin. Orthop. Relat. Res..

[21] Gardner J., Gioe T.J., Tatman P. (2011). Can This Prosthesis Be Saved?: Implant Salvage Attempts in Infected Primary TKA. Clin. Orthop. Relat. Res..

[22] Lizaur-Utrilla A., Asensio-Pascual A., Gonzalez-Parreño S., Miralles-Muñoz F.A., Lopez-Prats F.A. (2019). Negative impact of prior debridement on functional outcome of subsequent two-stage revision for early knee periprosthetic infection. Knee Surg. Sports Traumatol. Arthrosc..

[23] Brimmo O., Ramanathan D., Schiltz N.K., Pillai A.L.P.C., Klika A.K., Barsoum W.K. (2015). Irrigation and Debridement Before a 2-Stage Revision Total Knee Arthroplasty Does Not Increase Risk of Failure. J. Arthroplast..

[24] Nodzo S.R., Boyle K.K., Nocon A.A., Henry M.W., Mayman D.J., Westrich G.H. (2017). The Influence of a Failed Irrigation and Debridement on the Outcomes of a Subsequent 2-Stage Revision Knee Arthroplasty. J. Arthroplast..

[25] Öztürk Ö., Özdemir M., Turgut M.C., Altay M. (2021). The Fate of Failed Debridement, Antibiotics, and Implant Retention in Infected Knee Arthroplasties: Nothing to Lose. Cureus.

[26] Kim K., Zhu M., Cavadino A., Munro J.T., Young S.W. (2019). Failed Debridement and Implant Retention Does Not Compromise the Success of Subsequent Staged Revision in Infected Total Knee Arthroplasty. J. Arthroplast..

[27] Backstein D., Mont M., Krueger C., Browne J., Krebs V., Mason J.B., Taunton M., Callaghan J. (2020). Balancing Simplicity With Doing No Harm: The Case for Repeat DAIR Procedures. J. Arthroplast..

[28] McNally M., Sousa R., Wouthuyzen-Bakker M., Chen A.F., Soriano A., Vogely H.C., Clauss M., Higuera C.A., Trebše R. (2021). The EBJIS definition of periprosthetic joint infection. Bone Jt. J..

[29] R Core Team (2021). R: A Language and Environment for Statistical Computing.

[30] McQuivey K.S., Bingham J., Chung A., Clarke H., Schwartz A., Pollock J.R., Beauchamp C., Spangehl M.J. (2021). The Double DAIR: A 2-Stage Debridement with Prosthesis-Retention Protocol for Acute Periprosthetic Joint Infections. JBJS Essent. Surg. Tech..

[31] Estes C.S., Beauchamp C.P., Clarke H.D., Spangehl M.J. (2010). A Two-stage Retention Débridement Protocol for Acute Periprosthetic Joint Infections. Clin. Orthop. Relat. Res..

[32] Chung A.S., Niesen M.C., Graber T.J., Schwartz A.J., Beauchamp C.P., Clarke H.D., Spangehl M.J. (2019). Two-Stage Debridement With Prosthesis Retention for Acute Periprosthetic Joint Infections. J. Arthroplast..

[33] Antonios J.K., Bozic K.J., Clarke H.D., Spangehl M.J., Bingham J.S., Schwartz A.J. (2021). Cost-effectiveness of Single vs Double Debridement and Implant Retention for Acute Periprosthetic Joint Infections in Total Knee Arthroplasty: A Markov Model. Arthroplast. Today.

